# Driving force of deteriorated cellular environment in heart failure: Metabolic remodeling

**DOI:** 10.1016/j.clinsp.2023.100263

**Published:** 2023-08-07

**Authors:** Lu Fan, Chenchen Meng, Xiaoming Wang, Yunjiao Wang, Yanyang Li, Shichao Lv, Junping Zhang

**Affiliations:** a First Teaching Hospital of Tianjin University of Traditional Chinese Medicine, Tianjin, China; b National Clinical Research Center for Chinese Medicine Acupuncture and Moxibustion, Tianjin, China; c Tianjin Medical University Cancer Institute and Hospital, Tianjin, China; d Tianjin Key Laboratory of Traditional Research of TCM Prescription and Syndrome, Tianjin, China

**Keywords:** Heart failure, Physiological metabolism, Metabolic remodeling, Deteriorated cellular environment, Pharmacological agents, Stem cells

## Abstract

Heart Failure (HF) has been one of the leading causes of death worldwide. Though its latent mechanism and therapeutic manipulation are updated and developed ceaselessly, there remain great gaps in the cognition of heart failure. High morbidity and readmission rates among HF patients are waiting to be addressed. Recent studies have found that myocardial energy metabolism was closely related to heart failure, in which substrate utilization, as well as intermediate metabolism disorders, insulin resistance, oxidative stress, and mitochondrial dysfunction, might underlie systolic dysfunction and progression of HF. This article centers on the changes and counteraction of cardiac energy metabolism in the failing heart. Therefore, targeting impaired energy provision is of great potential in the treatment of HF. And shifting the objective from traditional neurohormones to improving the cellular environment is expected to further optimize the management of HF.

## Highlights

• Improving the cellular environment is expected to further optimize the management of HF.
• Metabolic remodeling is the driving force of deteriorated cellular environment in HF.
• Targeting impaired energy provision is of great potential in the treatment of HF.

## Introduction

HF is characterized by impaired ventricular filling and ejection function, which is the ultimate destination of numerous cardiovascular diseases [1]. It is estimated that the prevalence of HF in adults was about 1%‒2%, with over 13.7 million people suffering from HF worldwide. Thus, HF has brought about severe public health issues and financial burdens [2,3]. With the aging of the population, there is an upward trend in the incidence of chronic diseases at an earlier age, such as hypertension, Diabetes Mellitus (DM), and obesity, and the age of onset tends to be younger. The prompt precaution and management of cardiovascular diseases prolong the lifetime of patients, which leads to the great number of HF patients [4,5]. In addition, the high death and all-cause hospitalization rates remain the greatest threat to HF patients. The research reported that the readmission rates within 30 days amounted to 24.8%, and 1-year, 5-year, and 10-year survival rates were 86.5%, 56.7% and 34.9%, respectively [6,7]. The abnormal structure and function lead to disorders in blood circulation, appearing low cardiac output and fluid retention whose severity is consistent with symptoms and signs of HF. There is a general belief that myocardial remodeling is an adaptive change to hemodynamic abnormality. And the activation of the sympathetic nervous system as well as inflammatory response play a pivotal role that aggravates the development of ventricular remodeling [8]. Recent studies show that marked alterations of energy metabolism in the failing heart were going on. The concomitant insulin resistance, lipotoxicity, oxidative stress, and imbalance of calcium homeostasis worsen the cellular environment and drive the progression of the failing heart. Above all, targeting cardiac metabolism to optimize the cellular environment provides new possibilities for the treatment and management of HF.

### The physiological role of energy metabolism in the normal heart

The heart is a high-energy-consuming organ, accounting for approximately 12% oxygen consumption of the whole body [9]. And the heart never fails to utilize a range of substrates at hand for the sake of acclimatizing the changing environment. But very often, Fatty Acid Oxidation (FAO), meeting about 60% of the cardiac demand, is the main energy provision, and the rest suppliers include glucose, Ketone Bodies (KBs) and amino acids [10]. The fatty acid requires two transmembrane movements before oxidation in the mitochondria, and the membrane proteins involved in this process include Fatty-Acid Translocase (FAT/CD36), plasma membrane-associated Fatty-Acid Binding Protein (FABPpm), and Fatty-Acid Transport Proteins (FATP) [11]. Among them, it was observed in CD36(-/-) mice that fatty acid uptake mediated by CD36 accounted for 70% of total intake [12]. Peroxisome-Proliferator-Activated Receptor (PPAR), a ligand-activated nuclear transcription factor, positively regulates FAO involving uptake (FAT/CD36), storage (FABP), and Transport (CPT-1) in cardiomyocytes [13,14]. A recent study found that fatty acids excited the acetylation of CREB-binding protein-dependent Ovarian-Tumor-Domain-containing Deubiquitinase 3 (OTUD3) which adjusted relative genes referring to metabolism and oxidative phosphorylation by stabilizing PPARδ [15].

The uptake of cardiac glucose is determined by the concentration of glucose, the number and intrinsic activity of Glucose Transporter 4 (GLUT4) [16]. Glucose is phosphorylated to Glucose 6-Phosphate (G6P) by hexokinase, and glycolysis is the preferred destination for G6P [17]. The coupling between glycolysis and ion transport ensures the optimal transmission of energy between ion channels and transporters in cellular activities, which play a vital role in maintaining the systolic function, though glycolysis does not contribute much in terms of energy production [18]. Glucose and fatty acids regulate each other in a competitive way to maintain metabolic homeostasis, namely the Randle cycle. It is generally believed that pyruvate dehydrogenase complex and acetyl-CoA carboxylase are connected with the regulation of the Acetyl-CoA which is the coincidence and competition point of metabolism [19].

**Fig. 1:**
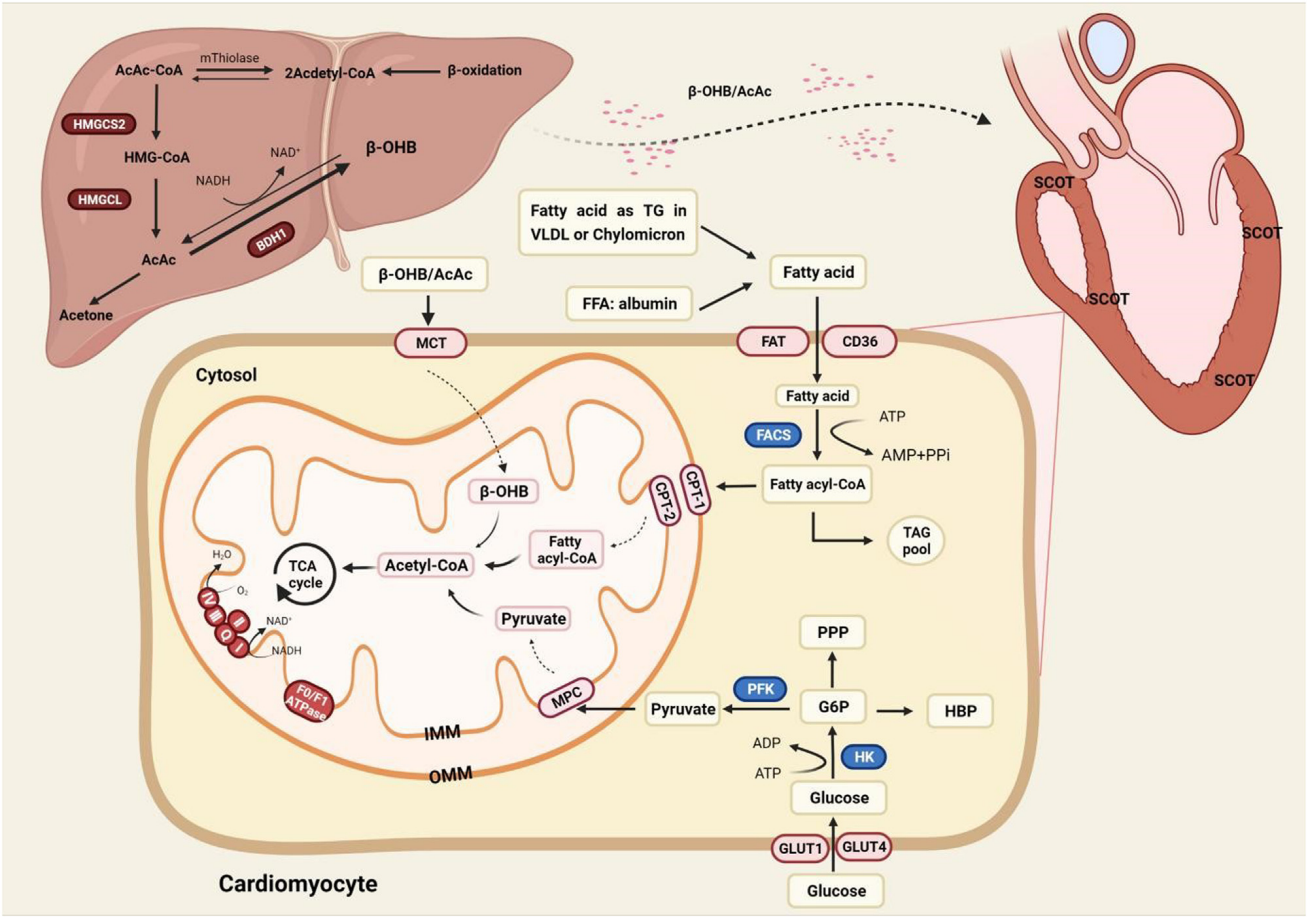
The energy metabolism in the normal heart. Fatty acid oxidation occupies center stage in energy provision, and the rest suppliers include glucose and ketone bodies. KBs can only be metabolized outside the liver as the liver is short of crucial enzyme SCOT. AcAc, Acetylacetic Acid; BDH1, 3-Hydroxy-n-Butyrate Dehydrogenase; β-OHB, Beta-Hydroxybutyrate; CPT1/2, Carnitine Palmitoyltransferase-1/2; FACS, Fatty Acyl CoA Synthetase; FAT, Fatty Acid Translocase (also known as CD36); FFA, Free Fatty Acid; GLUT1/4, Glucose Transporter 1/4; G6P, Glucose 6-Phosphate; HBP, Hexosamine Biosynthesis Pathway; HMGCL, 3-Hydroxy-3-Methylglutaryl-CoA Lyase; HMG-CoA, 3-Hydroxy-3-Methylglutaryl-CoA; HMGCS2, 3-Hydroxy-3-Methylglutaryl-CoA Synthetase 2; MCT, Medium-Chain Triacylglycerol; MPC, Mitochondrial Pyruvate Carrier; PFK, 6-Phosphofructokinase; PPP, Pentose Phosphate Pathway; SCOT, Succinyl CoA: 3-oxoacid-CoA-Transferase; TAG, Triacylglyceride; TCA cycle, Tricarboxylic Acid; VLDL, Very Low-Density Lipoprotein.

KBs include Acetylacetic Acid (AcAc), β-Hydroxybutyrate (β-HB), and acetone. KBs provide only about 5% needs of the whole body, and the contribution of KBs can rise up to 20% during fasting [20]. Ketone bodies, the intermediate of β-oxidation, are mainly synthesized in the mitochondria of the liver via 3-Hydroxy-3-Methylglutaryl-CoA (HMG-CoA) ketogenesis pathway. Ketone bodies are metabolized extrahepatic as a transport form of acetyl-CoA, catalyzing the exchange of CoA between succinic acid and AcAc. Besides, KBs also act as a signal transducer factor to regulate oxidative stress and the post-translational modification of protein [21] (Fig. 1).

### The pathologic change of energy in the failing heart

The process of HF is accompanied by poor energy production and metabolic remodeling. Metabolic remodeling involves increased neurohormonal stimulation and impaired calcium processing that exacerbate energy expenditure. These changes disrupt the balance between energy supply and demand, challenging the cell environment and promoting cardiomyopathy.

### Disorders of cardiac metabolism in the failing heart

#### Variations in the utilization of the fatty acid

Metabolic changes seem complex in failing hearts, not only depend on the severity and type of HF, but also highly correlated with comorbidities, such as obesity and Type 2 Diabetes (T2D). Although the exact metabolic changes and substrate preferences in HF remain controversial, current studies believe that FAO is unchanged or slightly increased in the early stage of HF and significantly decreased in later (Tables 1 and 2). Studies have shown that there was impaired microcirculation in obesity and diabetes. And Advanced Glycation Endproducts (AGEs) activate inflammatory signals and mediate cell apoptosis and fibrosis [22]. Meanwhile, molecular variations such as transcription, post-translational modifications, and mitochondrial biogenesis contribute to the diversity of pathological energy metabolism in HF [23]. PPAR plays a central role in regulating FA metabolism. There were declined FAO and UCP3 expression followed by heart failure in PPARα-deficient mouse models [24]. On the other hand, overexpression of PPARαbrings about cardiac dysfunction on account of the mismatch between FA uptake and utilization [25]. Estrogen-related Receptors (ERRs), orphan nuclear receptors, take part in the regulation of genes referring to mitochondrial energy transduction, systolic function, and ion transport. A study found that ERRα/γ knockdown resulted in the arrest of mitochondrial maturation, activation of fibroblast-related genes, and eventually developed heart failure [26]. Interestingly, overexpression of ERRγ in mice also showed signs of cardiac hypertrophy and fibrosis in a GATA4-driven manner [27]. However, the relationship between cardiac function and PPAR as well as ERR expression in the failing heart has not been specifically studied.

**Table 1:** Study relevant to cardiac metabolism changes in human heart failure.

**Population**	**Baseline**	**Main findings**	**Ref.**
**Fatty acid**			
HF (n = 8)	chronic, severe AHA/ACC Stage D HF	Decreased abundance of medium to long-chain acylcarnitines in the failing hearts;	[34]
		Decreased succinyl-CoA and increased acetyl-CoA in end-stage failing myocardium	
HF (n = 18)	A 10-month history of IDCM;	Unchanged oxidative metabolism in HF;	[114]
	Mean NYHA class: 2.2	Significantly decreased efficiency of patients	
CHF (n = 12)	Stable NYHA class III;	Higher myocardial fatty acid uptake in patients with HF;	[115]
	LVEF ≤35%	Elevated serum FFA	
HF (n = 19)	A 10-month history of IDCM;	Reduced myocardial FFA metabolism;	[116]
	Mean NYHA class: 2.2		
HFrEF (n = 15)	Mean age: 71 ± 6 years;	Unimpaired FFA oxidation;	[78]
	LVEF: 28 ± 8%	Significantly increased cardiac uptake of ketone bodies	
**Glucose**			
non-ischemic HF (n = 8)	Mean age: 40.5 ± 11.5 years;	Diminished glucose metabolism in failing hearts;	[117]
	Failing heart: LVEF 10%;	Recovered their values post-LVAD	
	Post-LVAD heart: LVEF 26%		
CHF (n = 10)	Mean age: 60.6 ± 1.1 years;	Impaired glucose handling;	[80]
	NYHA class ≥ II	Decreased insulin-mediated stimulation of total-body oxidative glucose metabolism	
**Ketone body**			
HF (n = 46)	LVEF: 39.59 ± 10.94%;	Significantly increased hydroxybutyrate and acetone levels	[80]
	NYHA class ≥ II		
HF (n = 615)	Low acetoacetate group (n = 302): LVEF 52 ± 16%;	High levels of circulating acetoacetate were associated with older age, higher NYHA classification, hypertension, high B-type natriuretic peptide levels, and worse clinical outcome.	[118]
	High acetoacetate group (n = 313): LVEF 50 ± 16%		

CHF, Congestive Heart Failure; FFA, Free Fatty Acid; HF, Heart Failure; HFrEF, Heart Failure with reduced Ejection Fraction; IDCM, Idiopathic Dilated Cardiomyopathy; LVAD, Left Ventricular Assist Device; LVEF, Left Ventricular Ejection Fraction; NYHA, New York Heart Association.

**Table 2:** Study relevant to cardiac metabolism changes in rats models of heart failure.

**Animal model**	**Intervention**	**Events**	**Cardiac outcome**	**Ref.**
**Fatty acid**				
Rats with post-infarction HF	CAL	Markedly decreased myocardial mRNA expression of H-FABP and MCAD	Cardiac compensated remodeling	[119]
DS rats with CHF	TAC	Decreased gene expression of PPARα, PGC1-α and fatty acid transporter	Compensated LVH;	[120]
	Fed a high-salt diet		Decreased cardiac PCr/ATP	
C57BL/6 mice with HF	TAC	Rapid normalization of cardiac oxidative metabolism	Cardiac fibrosis;	[121]
			Severe exercise intolerance	
C57BL/6N mice with HF	TAC	Elevated rates of systemic FA utilization	Cardiac fibrosis;	[122]
			Exercise intolerance	
C57BL/6 mice with HF	Post-infarction (MCD-KO & CAL)	Almost unchanged rates of glycogen degradation, glycogen synthesis, TG degradation and synthesis	Systolic dysfunctions;	[123]
			Reduced LVEF	
Obese C57BL/6J mice with HF	AAC	FAO dominated as source of ATP production	Cardiac insulin resistance;	[124]
	Fed a high-fat diet		Developed diastolic dysfunction	
**Glucose**				
C57BL/6 mice with HF	AAC	Reduction insulin-stimulated glucose utilization;	Marked cardiac insulin resistance;	[32]
		Impaired plasma membrane translocation of GLUT4;		
		Elevated glycolysis	Developed diastolic dysfunction	
C57/Bl6 mice with HF	TAC	Reduced rates of glucose and lactate oxidation;	Systolic dysfunction;	[125]
		Unchanged glycolysis or FAO	Diastolic dysfunction	
DS rats with HFpEF	Fed a high salt diet	Increased glycolysis;	Cardiac hypertrophy;	[126]
		Unchanged glucose oxidation rates;	Diastolic dysfunction	
		Increased GLUT1 expression		
DS rats with CHF	Fed a high-salt diet	Increased glucose uptake and decreased fatty acid uptake;	Concentric LVH;	[120]
		Decreased expression of GLUT4 and increased GLUT1	Cardiac dysfunction	
**Ketone body**				
C57BL/6J mice with HF	TAC & post-infarction	Increased expression of BDH 1;	Reduced left ventricular systolic function;	[33]
		Decreased gene expression involved in FA utilization;		
		Accumulated mitochondrial ketone oxidation	Global chamber dilatation	
C57BL/6J mice with HF	AAC	Elevated βOHB level;	Cardiac dysfunction;	[127]
		Down-regulated SCOT	Adverse cardiac remodeling	

AAC, Abdominal Aortic Constriction; BDH 1, β-Hydroxybutyrate Dehydrogenase 1; βOHB, β-Hydroxybutyrate; CAL, Coronary Artery Ligation; CHF, Congestive Heart Failure; DS, Dahl Salt-Sensitive; GLUT1/4, Glucose Transporter 1/4; FA, Fatty Acid; FAO, Fatty Acid Oxidation; HF, Heart Failure; H-FABP, Heart-Fatty Acid Binding Protein; HFpEF, Heart Failure with preserved Ejection Fraction; LVEF, Left Ventricular Ejection Fraction; LVH, Left Ventricular Hypertrophy; MCAD, Medium-Chain Acyl-CoA Dehydrogenase; MCD-KO, Knockout of Malonyl CoA Decarboxylase; MI, Myocardial Infarction; mRNA, Messenger RNA; PGC1-α, Peroxisome Proliferator-activatedreceptor-γ Coactivator-1α; PPARα, Peroxisome Proliferator-Activated Receptor alpha modulator; SCOT, Succinyl CoA:3-oxoacid CoA Transferase; TAC, Transverse Aortic Constriction; TG, Triglyceride.

#### Uncoupling between glucose oxidase and glycolysis

Increased glucose uptake and glycolysis are conspicuous marks of a failing heart, but glucose oxidation is not synchronized with the increase in glycolysis [28,29]. When the heart is faced with overloaded pressure, the expression of PFK1 and Fructose 2,6 Bisphosphate (F2,6BP) are boosted which contributes to the uptrend flow through glycolysis in the failing heart [30]. On the other side, the impaired liveness of the rate-limiting enzyme of glucose oxidation in the failing heart may aggravate the mismatch. The analysis of metabolomics, gene transcripts, and proteomics of tissues from the left ventricle revealed that mRNA expressions of PDH, MCT1, and pyruvate/alanine aminotransferase were reduced in HF. The possible mechanism is that hyperacetylation induced by HF and the hyperacetylation of PDH may inhibit their activity [31]. The reduction of myocardial glucose oxidation precedes the onset of diastolic dysfunction in hypertrophy mice which further verifies the adaptive role of reduced glucose oxidation in the development of heart failure [32].

#### Increased contribution of ketone bodies

Emerging evidence suggested that under the condition of low cardiac output, increased lipolysis in HF patients augmented the availability of ketone bodies, and overexcitation of the sympathetic nervous system promoted the generation and utilization of KBs (Tables 1 and 2). In HF rat models, the expression of BDH1, catalyzing ketone body oxidation, was elevated. And the same phenomenon was observed in patients with end-stage HF [33,34]. It is generally accepted that KBs are more efficient superfuels than other substrates [35]. In fact, recent studies have challenged the notion that ketone bodies are fuel-saving for the failing heart. Research indicated that the additional reducing equivalent accompanied by ketone bodies oxidation didn't match the production of ATP. The findings suggested that elevated ketone oxidation in the failing heart didn't do good to cardiac efficiency though KBs prompt ATP synthesis [36,37]. Therefore, the role of ketone body utilization in the development of HF is waiting to be further confirmed.

### Dysfunction of mitochondria in the failing heart

Mitochondria are in charge of the homeostasis of energy metabolism. The latest study found that the expression of Dual-Specificity Tyrosine-Regulated Kinase 1B (DYRK1B) which mediated cardiac hypertrophy and fibrosis by damaging mitochondrial bioenergetics was upregulated in HF. DYRK1B increases its phosphorylation and nuclear accumulation by directly binding to STAT3, leading to the down-regulation of PGC-1α level and subsequent cardiac insufficiency [38]. Mitochondrial function is controlled by multiple post-translational modifications. In animal models and human failing hearts, acetylation of mitochondrial proteins was significantly increased which resulted in an impaired mitochondrial respiratory chain [39]. The reduced protein deacetylation is the latent mechanism. A study found that the expression of mitochondrial deacetylase SIRT3 was decreased and proved that Mir-195 down-regulated SIRT3 expression by directly targeting the direct 3′-untranslated region [40]. Conversely, the activity of deacetylation relies on the availability of NAD^+^. Hyperacetylation inhibits the activity of the malate-aspartate shuttle which is the limitation of the transport of NADH from cytoplasm to mitochondria, and then disrupts the cytoplasmic REDOX state of the failing heart [41].

## Counteraction of metabolic remodeling ‒ deteriorated cellular environment

### Insulin resistance

Failing hearts exhibit significant metabolic remodeling, and there is a debate about whether these alterations contribute to the development of heart failure. A study involving 2623 patients found that there were increased wall thickness and LV mass with the deterioration of glucose intolerance among patients [42]. Normally, insulin reduces mitochondrial FA uptake by increasing the activity of acetyl-CoA carboxylase. Impaired mitochondrial β-oxidation and boosted FAO rate are accompanied by changes in cardiac insulin signaling, including the activation of the proximal insulin signaling pathway IRS/Akt [32]. This alteration contributes to insulin resistance characterized by reduced glucose oxidation and impaired inhibition of FAO. Recent studies found that IRS-1/Akt1 was activated in the failing heart and the deficiency of IRS1 exerted a protective effect in HF mice while IRS-2 acted the opposite [43]. In a post-MI mouse model, it was found that the injured myocardium promoted the degradation of Insulin Receptor Substrate 1 (IRS1) by upregulating MIR128-3p [44]. However, scholars proposed that insulin resistance provided protection for myocardial fuel overload in obesity or diabetes [45].

### Lipotoxicity

In heart failure, the chaos of uptake and utilization of FA gives rise to the accumulation of lipid intermediates, such as ceramide and diacylglycerol. The study has found that the overexpression of GSK3α boosted the uptake of FFA in the failing heart leading to fibrosis and cardiac hypertrophy [46]. Similarly, increased FFA uptake was found to be accompanied by impaired left ventricular filling function and atrial enlargement in FATP1^+/+^ mice [47]. Under the circumstances, endoplasmic reticulum stress makes negative impact on cardiomyocytes mediated by pressure-driven lipid accumulation, which is correlated with the expression of the Very Low-Density Lipoprotein Receptor (VLDLR). Lipotoxicity, a byproduct of this process, may cause myocardial apoptosis, insulin resistance and systolic dysfunction [48]. This point of view was confirmed by reduced ischemia-induced endoplasmic reticulum stress and cardiac apoptosis among VLDLR^−/−^ mice and mice treated with antibodies specific for VLDLR [49]. The study has proved that the expression of Mst1 elevated in rats with lipid-induced heart injury. In this study, lipotoxicity induced the expression of Forkhead box O3 (FoxO3) by promoting the binding of FoxO3 to the Mst1 promoter, and deficiency of Mst1 gene ameliorated apoptosis and inflammation [50].

### Oxidative stress

The extra glucose entered pathways, mainly Pentose Phosphate Pathway (PPP), and Hexosamine Biosynthesis Pathway (HBP). Glucose 6-Phosphate Dehydrogenase (G6PD), the rate-limiting enzyme of PPP, is essential to maintain the REDOX state of cardiomyocytes. And elevated expression and activity of G6PD are observed in both humans and canines [51,52]. Studies showed that G6PD deficiency might contribute to cardiac dysfunction by boosting susceptibility to free radical damage and impairing intracellular ion transport [53]. As an adaptive response, HBP mediates the O-linked-β-N-Acetylglucosaminylation (O-GlcNAcylation) of proteins via glutamine-fructose-6-phosphate while the integration of various cellular signals, including intracellular and intracellular stress and nutrient levels [54,55]. Glutamine-Fructose-6-phosphate Transaminase (GFAT) is a key enzyme in HBP, and studies found that GFAT1 was the target of X-box binding protein 1 (Xbp1s), a pivotal transcription factor of Unfolded Protein Reaction (UPR). The overexpression of XBP1s in cardiomyocytes gives rise to elevated activity of HBP and O-GlcNAcylation [56]. Besides, oxidative stress also disrupts the Nitric Oxide (NO); Soluble Guanylate Cyclase (sGC); Cyclic Guanosine Monophosphate (cGMP) pathway, causing elevated cGMP levels in cells, further promoting the development of HF [57].

### Calcium dyshomeostasis

Ca^2+^, governing EC coupling, is the bridge of the communication between cardiac electrical activity and excitation-contraction coupling. In HF, Ca^2+^ leaks through the channel RyR2. Current research has identified several potential factors for Ca^2+^ leakage, including the conformational change of RyR2 induced by hyperphosphorylation [58,59]. In addition, abnormal mitochondrial aggregation and structural reorganization may impair the transportation of Ca^2+^ signaling, and then give rise to ROS accumulation [60]. ROS, a by-product of mitochondrial respiration, has a negative impact on the failing heart related to the activation of multiple signaling pathways and apoptosis. Studies confirmed that Calcium/Calmodulin-dependent protein Kinase II (CaMKII) took a vital part in the development of HF that promoted mPTP opening and apoptosis by mediating the current of the inner membrane Mitochondrial Ca^2+^ Uniporter (MCU) [61,62]. Latest research indicated that ROS-mediated oxidation induced the changes in mitochondrial permeability which led to cellular damage and apoptosis by activating RIP3, an upstream kinase of CaMKII [63]. To a certain degree, cardiac function relies on the persistent supply of ATP and the balance between ROS production and clearance. In HF and other cardiac diseases, EC uncoupling and impaired mitochondrial Ca^2+^ dynamics are widespread that deteriorate the cellular environment.

### Redistribution of receptors on cardiomyocytes

Heart failure causes significant changes in hormone receptors, which typically manifests as the failing heart escaping from the supervision of adrenergic. And the downregulation of β1 receptor is the most significant [64]. Myocardial membrane biopsy of HF patients indicated that the density of β-AR gradually decreased as the increased severity of heart failure [65]. Meanwhile, the ratio of β1:β2-AR decreased from 80:20 to 60:40, and other changes included increased β-AR uncoupling and G protein activity [66]. β-AR receptors are dominant in the human heart, among which β3-AR is closely related to glucose and lipid metabolism. It is suggested that β3-AR confronts the myocardial contraction through NO/cGMP pathway. Under physiological conditions, the level of β3-AR in myocardial tissue is extremely low (only 3%), and the expression of β3-AR is significantly upregulated in failing hearts and shows a myocardial protective effect [67,68]. Clinical studies have shown that mirabegrone (the β3-AR receptor agonist) improved insulin sensitivity and decreases plasma hemoglobin A1c levels in obese patients, and the expression of PGC1, transcription factor A, and cyclooxygenase IV was increased. In addition, Mirabelone can increase the level of Nonesterified Fatty Acids (NEFA) in plasma and adipose tissue. And it is widely believed that a high level of NEFA contributes to ectopic lipid accumulation and the inhibition of insulin receptor signaling in skeletal muscle [69]. β3-AR agonist improved LVEDP and end-systolic pressure-volume relation slope, reducing ventricular muscle stiffness and inhibiting Smad2/3, phospho-Smad2/3, and TGF-β1 expression in CHF mice [70]. Overexpression of β3-AR relieved cardiomyocyte hypertrophy, delaying the progression of heart failure, reducing mitochondrial fragmentation, and maintaining mitochondrial ridge integrity and dynamic homeostasis in Aortic stenosis mice [71]. Intracellular Ca^2+^ is a major factor affecting arrhythmia during heart failure. β3-AR agonists can reduce the incidence of ventricular arrhythmia in CHF rabbits, and inhibit INCX, Ca^2+^ transient, SR Ca^2+^ load and leakage [72]. Whereas, the absence of β3-AR receptor caused left ventricular diastolic dysfunction in mice [73]. PPAR is involved in the regulation of blood cholesterol and glucose concentration, among which PPARα/δ is the dominant subtype in the heart tissue. In HF, increased adrenergic tension activates signal transduction molecules such as HIF, mTOR, and NF-κB, thereby reversely inhibiting the expression and activity of PPARα/δ, which results in a shift in the energy metabolic profile of the failing heart to the infantile pattern [74]. P2 purinergic receptors are key molecules for ATP. The expression of P2X4/7 is the highest in the human sinoatrial node. In MI-induced HF rats, the mRNA expression of P2X4 was elevated in the sinus node. And the highly expressed of P2X4 receptor increased the formation of S-nitrosylation, cGMP, and NO in hyperlipidemia mice [75,76]. The expression of P2Y6 receptor was elevated in TAC mice and induced fibrosis through the activation of Galpha 12/13 [77]. However, the relationship between changes in receptors of cardiomyocytes and heart failure has not been specifically studied.

### Metabolic changes in different types of heart failure

In heart failure, the pathophysiological differences of different types of HF make the uptake and utilization of energy substrates present different metabolic profiles. A study collected the coronary blood samples of 15 patients with HFrEF. The results indicated that compared with the control group, the absorption of FFA and glucose in HFrEF patients had not increased, while the plasma β-hydroxybutyrate and acetylacetate levels were slightly elevated, and the concentration of multiple acylcarnitines increased. In terms of energy contribution, the relative contributions of FFA and glucose decreased, while the relative contributions of ketone bodies increased. And the contribution of beta-hydroxybutyric acid and acetoacetic acid on the ATP production increased from 6.8% in the control group to 24% in the HFrEF group [78]. Another study included 46 patients with decreased ejection fraction (LVEF: 39.59 ± 10.94%; NYHA class ≥ II) and showed that the concentration of 3-hydroxybutyrate, acetone and succinate were significantly elevated with the increase of cardiac energy consumption [79]. 82 serum samples were quantitatively detected by LC-MS/MS and 1H-NMR. The results showed that compared with the non-HF group, the serum concentrations of acylcarnitine, carnitine, creatinine, betaine, and amino acids in HFpEF patients were increased, while the levels of phosphatidylcholine, lysophosphatidylcholine, and sphingomyelin were decreased. However, the levels of medium and long-chain acylcarnitine and ketone bodies in HFpEF patients were higher than those with HFrEF [80]. Cardiac magnetic resonance was applied to detect the myocardial fat content in HFpEF (n = 163), HFrEF (n = 34) and the control group, and the results showed that the cardiac fat content in the HFpEF group was more than twice that of the control group. On the contrary, the HFrEF group showed a downward trend [81]. In addition, most studies support the idea that under pressure overload the utilization of glucose increases. Positron emission tomography showed that there were increased glucose uptake and utilization, and decreased FFA among patients with cardiomyopathy [82]. Basic studies have shown that the glucose uptake and utilization increased on the first day after TAC operation in HF mice, and showed an elevated trend with time [83]. The changes in energy metabolism in a failing heart are complex, and the severity, type, stage, and comorbidities of heart failure will have a profound impact on the utilization of heart energy.

## Treatments

### Pharmacological agents for improving the cellular metabolic environment

#### Altering fatty acid oxidation

Whether the reduction in FAO during HF is either protective or unadapted determines administration of medication. Based on what boosted FAO is strongly correlated with the increase of ROS, inhibition of FAO may exert a great cardioprotective effect by inhibiting intracellular oxidative stress levels, preventing the accumulation of toxic lipid intermediates, and maintaining cellular environmental homeostasis [84]. Trimetazidine and ranolazine, optimizing substrates of myocardial metabolism, are inhibitors of 3-Ketoacyl coenzyme A Thiolase (3-KAT) which catalyzes the last step of β-oxidation. Trimetazidine indirectly stimulates the activity of PDH and raises the utilization of glucose by selectively inhibiting FAO [85]. In mouse models with HF induced by pressure-overloading, trimetazidine activated AMPK in a dose-dependent manner to enhance glucose uptake as well as transformation of metabolic substrates and ameliorate insulin resistance [86]. And trimetazidine decreases the accumulation of H^+^ and lactic acid in the cytoplasm, in turn, avoids calcium overload and other adverse cardiac events [87]. Renolazine alleviates myocardial hypertrophy and fibrosis by optimizing myocardial energy metabolism and alleviating Na+-dependent calcium overload [88]. β-blockers are the cornerstone drugs in the treatment of heart failure, which can shift myocardial substrate utilization from FFA to glucose oxidation, thereby reducing myocardial oxygen consumption and improving myocardial efficiency. In 26 patients with moderate to severe heart failure treated with carvedilol for 6 months, the lipid oxidation rate decreased significantly (2.4±1.4 to 1.5±0.9 mg m^2^/kg min) [89]. Glucose oxidation rate increased (2.6±1.4 to 4.4±1.6 mg m^2^/kg min). Basic experiments showed that metoprolol alleviated cardiac dysfunction, reducing palmitate oxidation rate, stimulating glucose oxidation, and increasing tissue ATP levels in diabetic rats [90]. While recent studies have shown that II/III generation β-blockers have little effect on substrate oxidation among HFrEF patients [91].

Besides, reducing cardiac FAO through the modification of PPARs is a latent therapeutic approach. Fibrates, the PPARs modulators, decrease β-oxidation by reducing FFA levels and increasing the utilization by other tissues. Studies confirmed that pemafibrate induced the expression of Lipoprotein Lipase (LPL) in mice by activating PPARα to reduce TG and lessen the secretion of LDL-C. At the same time, based on the concept of easing the burden on kidneys, pemafibrate is mainly metabolized through the liver, and excretion through urine only accounts for 14.5%. Thus, pemafibrate can be safely applied in patients with chronic kidney disease [92]. The accumulation of ROS and toxic lipid metabolites caused by FFA is a vital agent for the deteriorated cellular environment. However, there is a potential risk of reducing fatty acid oxidation, which would further reduce the energy supply of ATP. Therefore, the management of heart failure through inhibiting fatty acid oxidation needs to be carefully considered.

#### Modulating glucose oxidation

Insulin signaling is an important metabolic pathway in the cellular environment. In heart failure, neurohumoral and cytokine imbalances and cellular oxidative stress induce insulin resistance, resulting in increased fatty acid flux into cardiomyocytes. Since glucose is a more efficient substrate, the switch in cardiometabolic metabolism from glucose utilization to fatty acid oxidation may reduce cardiac efficiency. In the severe stage of heart failure, ATP level declines sharply and the inhibition of FAO may further damage cardiac function. Thus, direct stimulation of glucose oxidation may be a better option. Metformin was verified to have a relation with reduced HF mortality as well as readmission in a meta-analysis, with a 22% lower all-cause mortality for patients taking metformin than for those not [93]. Studies found that Metformin promoted the uptake as well as utilization of glucose and lowered FFA to avoid the lipotoxicity followed by FA accumulation. Other beneficial effects include improving the function of vascular endothelial cells and fighting against oxidative stress [94]. A recent experiment revealed the direct molecular target of metformin. Metformin-bound PEN2 forms a complex with ATP6AP1, a subunit of the v-ATPase8, which induces the inhibition of v-ATPase and the activation of AMPK [95]. The process enhances that metformin performs its beneficial role without substantial side effects.

2021 ESC Guidelines clearly brought Sodium-Glucose cotransporter-2 inhibitors (SGLT2i) as the primary drug for the treatment of heart failure with reduced ejection fraction (HFrEF). Its latent beneficial effects on the heart are as follows: Firstly, SGLT2i, acting like osmotic diuretics, improves ventricular load conditions, optimizes volume management, and reduces cardiac energy consumption by boosting sodium and glucose excretion [96]. Secondly, SGLT2i may transform the utilization of myocardial substrates and then boost the production and storage of ATP in mitochondria on account of promoting the decomposition of FA and lifting the level of KBs [97]. Thirdly, the newest research found that SGLT2i presented cardioprotective effects by regulating excessive autophagy of myocardium, directly suppressing the activity of the Na^+^/H^+^ Exchanger 1 (NHE1) in the cardiomyocytes [98]. A recent trial involving 4744 patients with HFrEF confirmed that the application of dapagliflozin lowered the risk of worsening heart failure or death from cardiovascular causes more than those who received a placebo, regardless of the presence or absence of DM [99]. And studies have verified that facilitating cardiac ketone body oxidation was the protective way against the failing heart. The application of ketone ester in a post-MI rat might improve cardiac function and ameliorate cardiac remodeling by reprogramming the genetic expression involved in KBs utilization [100]. However, it seems unable to maintain high circulating KBs level in the long term, whereas the emergence of SGLT2i, increasing ketone bodies to support TCA circulation, overcomes this problem. And other studies showed that increased circulating ketone bodies following the administration of SGLT2i may relieve inflammation in the failing heart by attenuating NLRP3 inflammasome activation [101]. It is worth noting that in the cellular environment, the advantages and disadvantages of a single metabolic substrate are not absolute. And the ability to maintain metabolic flexibility and boost ATP production plays an equally vital role in the diseases.

#### Improving mitochondrial dysfunction

Mitochondrial dysfunction has a close relation with oxidative stress during the development of HF, and attempts targeting mitochondrial ROS have shown advantages in the treatment of HF. Coenzyme Q, a part of the electron transport chain, is involved in electron transfer in ETC to regulate substrate oxidation and thereby exert antioxidant effects by removing excess ROS. Thus Coenzyme Q10 (CoQ10) supplementation has turned into a safe and effective option [102]. A meta-analysis incorporating 14 RCT experiments confirmed that the application of CoQ10 reduced mortality and improved the exercise capacity of patients with HF [103]. NAD^+^/NADH, at a relatively low-level amount patients with HF, is another prospective approach to restore the metabolic balance in the failing myocardium [104]. NAD^+^, the electron donor, participates in glycolysis, TCA cycle and oxidative phosphorylation while acting as a signal transduction molecule to regulate acetylation of mitochondrial proteins [105]. In a murine model with HFpEF, NAD^+^ repletion has been demonstrated to improve the mitochondrial function as well as exercise capacity and ameliorate the HFpEF phenotype [106]. This experiment supports the positive side of elevated NAD^+^ levels in the failing heart, but the efficacy and safety of exogenous NAD^+^ supplement therapy for HF need to be verified in clinical trials (Table 3).

**Table 3:** Pharmacological therapies targeting cardiac metabolism in heart failure.

**Agent**	**Mechanism of action**	**Drug class**	**Objects**	**Key results**	**Ref.**
Trimetazidine	FAO inhibition	LC3-KAT inhibitor;	HF (n = 955)	Significantly reduced left ventricular end-systolic volume;	[128]
				Improved NYHA class	
		Weak CPTI inhibitor	HF (n = 44)	Improved NYHA class and quality of life;	[129]
				Reduction in whole body REE	
Ranolazine	FAO inhibition	LC3-KAT inhibitor	HFpEF (n = 20)	Significantly decreased LVEDP vs. placebo;	[130]
				Improved measures of hemodynamics	
			CHF (n = 109);	Significantly improved LVEF;	[131]
			NYHA class: II‒IV	Decreased SB	
Pemafibrate	FAO stimulation	SPPARM-α	T2D (n ≈ 10,000)	On going (PROMINENT)	[92]
			ApoE2KI mice;	Exerting beneficial effects on FAO, RCT and inflammation	[132]
			HapoA-I tg mice		
Metformin	Glucose oxidation stimulation	Hypoglycaemic agent	HF (n = 34,504)	Small reduction in all-cause hospitalization;	[133]
				Reduced mortality;	
			CKD (n = 5);	Reduced HF readmission in patients with CKD or CHF;	[93]
			CHF (n = 11);		
			CLD (n = 3)	Reduced all-cause mortality	
Empagliflozin	Enhanced renal glucose excretion;	SGLT2i	HF (n = 3730);	Reduced risk of cardiovascular death or hospitalization for HF	[134]
			NYHA class: II‒IV		
Dapagliflozin	Increased circulating KB and FFA		HFrEF (n = 4744)	Reduced risk of cardiovascular death or worsening HF	[135]
Sotagliflozin		SGLT1/2i	Worsening HF (n = 1222)	Reduced risk of cardiovascular death or worsening HF	[136]
Mito Q	Mitochondrial ROS scavenging	Selective mitochondria targeted antioxidant	HF (n = 2149)	Reduced mortality;	[103]
				Improved exercise capacity	
			HF (n = 420)	Reduced cardiovascular mortality, all-cause mortality and incidence of hospital stays for HF;	[137]
				Significantly improved NYHA class	
NR	Improving mitochondrial function	NAD^+^ Repletion	C57BL/6N mice with HFpEF	Alleviated mitochondrial dysfunction;	[106]
				Improved cardiac function	
			Stage D HF (n = 19)	Improved mitochondrial respiration;	[138]
				Alleviated proinflammatory activation of PBMCs	

AMPK, AMP-Activated Protein Kinase; CHF, Congestive Heart Failure; CKD, Chronic Kidney Disease; CLD, Chronic Liver Disease; CPT I, Carnitine Palmitoyltransferase I; DM, Diabetes Mellitus; FAO, Fatty Acid Oxidation; FFA, Free Fatty Acid; HFpEF, Heart Failure with Preserved Ejection Fraction; KB, Ketone Bodies; LAV, Left Atrial Volume; LC3-KAT, Long-Chain 3-ketoacyl-CoA thiolase; LVEDP, Left Ventricular end-Diastolic Pressure; LVH, Left Ventricular Hypertrophy; LVEF, Left Ventricular Ejection Fraction; MCD, Malonyl-CoA Decarboxylase; MCD-KO, Knockout of Malonyl CoA Decarboxylase; MI, Myocardial Infarction; NR, Nicotinamide Riboside; NT-pro BNP, N-Terminalpro-B-type Natriuretic Peptide; PBMCs, Peripheral Blood Mononuclear Cells; PDK, 3-Phosphoinositide-Dependent Protein Kinase; PROMINENT, Rationale and Design of the Pemafibrate to Reduce cardiovascular outcomes by reducing triglycerides in patients with diabetes study; RCT, Reverse Cholesterol Transport; REE, Resting Energy Expenditure; ROS, Reactive Oxygen Species; SB, Sympathovagal Balance; sGC, Soluble Guanylate Cyclase; SGLT2i, Sodium-Glucose co-Transporter 2 inhibitors; SPPARM-α, Selective Peroxisome Proliferator-Activated Receptor alpha Modulator; T2D, Type 2 Diabetes mellitus.

### Stem cells targeting cytothesis

Injured cardiomyocytes enter the new cell cycle, and some endogenous cardiac stem cells may participate in the process of regeneration and repairment by regulating the secretion of cytokines. In the last decade, several types of stem cells have been applied to repair damaged cardiomyocytes in pre-clinical and clinical trials. For now, the hypotheses about latent mechanisms include promoting cardiomyocyte regeneration, angiopoiesis, reducing apoptosis, and intervening in ventricular remodeling by paracrine [107,108]. Studies found that injection of bone marrow Mesenchymal Stem Cells (MSCs) significantly elevated left ventricular ejection fraction, lessened end-systolic volume, and increased 6-minute walking distance without cardiotoxicity [109]. In a study involving 65 patients, MSCs infusion increased the expression of hepatocyte growth factors such as myogenesis, cell migration, and immune adjustment with improvements in the New York Heart Association class and the Minnesota Heart Failure Living Questionnaire under the standard treatment of heart failure [110]. Ixmyelocel-T consists of a mixture of cells including macrophages, granulocytes, monocytes, lymphocytes, and MSCs. In a study, the application of ixmyelocel-T in symptomatic HF patients realized a 37% reduction in adverse cardiovascular events compared with the control group [111].

Whereas, dysregulation of glucose metabolism may interfere with the positive effect of stem cells on heart failure. Among diabetic patients, CD26/DPP-4 on CD34^+^ cells failed to increase, suggesting that abnormal glucose metabolism and diabetes might impair the responsiveness of stem cells [112]. In another research, CD34^+^ stem cells failed to take effect among patients with diabetes, while putting up great responsiveness in patients with insulin resistance, implying that the effect of stem cell therapy might be regulated by glucose metabolism [113]. Therefore, it will be a new challenge to explore the factors that intervening in the efficacy of stem cells in the treatment of heart failure.

## Conclusion

A sound heart can make adaptive adjustments to cope with environmental changes according to the availability of the substrate. In heart failure, transcription of key enzymes involved in cardiac metabolism, REDOX, and changes in signal transduction give rise to cardiac disturbance of energy metabolism and impairment of metabolic flexibility. And put up with a harder challenge for survival. Hence, A better and more thorough understanding of the role and regulatory mechanisms of cardiac metabolism remodeling in HF may pave the way for the resolution of HF. Existing studies have proved that regulation of substrate utilization, oxidative phosphorylation, mitochondrial function, and cytothesis improved cardiac function and the quality of patients’ life. Taking all these factors into account, shifting the emphasis of pharmacotherapy from neurohormonal therapy to metabolic regulation is expected to be a key point in reducing the high readmission rate of HF. For decades to come, research ought to focus on clarifying the efficacy and safety of improving the cellular metabolic environment and seek systematic and feasible therapeutic regimens.

## Authors’ contributions

Lu Fan wrote the paper draft. ChenChen Meng and Xiaoming Wang drew the figures. Yunjiao Wang and Yanyang Li drew the tables. Shichao Lv and Junping Zhang corrected the draft. All authors agree to be accountable for all aspects of work ensuring integrity and accuracy. The authors declare that all data were generated in-house and that no paper mill was used.

## Declaration of Competing Interest

The authors declare that they have no known competing financial interests or personal relationships that could have appeared to influence the work reported in this paper.
